# Supplementary material for: Polyelectrolyte Functionalisation of Track Etched Membranes: Towards Charge-Tuneable Adsorber Materials
Source: Membranes (Basel). 2021 Jul 6;11(7):509. doi: 10.3390/membranes11070509 (PMC8304886; doi:10.3390/membranes11070509)
Supplement: Supplementary file 1 [file membranes-11-00509-s001.zip › membranes-1286093-supplementary.pdf]

Supporting Information

"Polyelectrolyte functionalisation of track etched membranes: Towards charge-tuneable adsorber materials"

Lisa Wiedenhöft[1], Mohamed M. A. Elleithy[2], Mathias Ulbricht[2] and Felix H. Schacher*,[1]

1: Institute of Organic Chemistry and Macromolecular Chemistry (IOMC) and Jena Center for Soft Matter (JCSM), Friedrich Schiller University Jena, 07743 Jena, Germany

felix.schacher@uni-jena.de

2: Lehrstuhl für Technische Chemie II, University of Duisburg-Essen, 45141 Essen, Germany

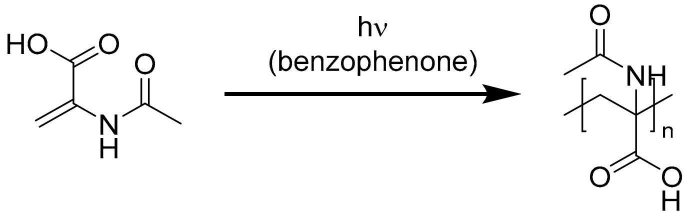

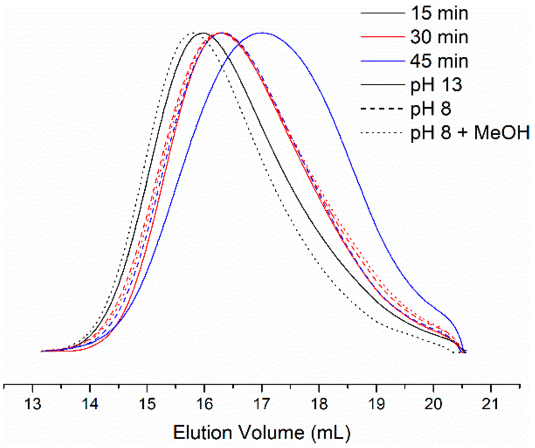

Figure S1: UV initiated free radical polymerisation of NADha: Changes in SEC elution traces for polymers with different irradiation times.

The surface area of the base-membranes was determined *via* the adsorption of krypton at 77 K. Using the BET-isotherm, a specific surface of 5.425 $m^2 \cdot g^{-1}$ (membrane with the average pore diameter of 0.2 µm) and 1.353 $m^2 \cdot g^{-1}$ (membrane with an average pore size of 1 µm) respectively.

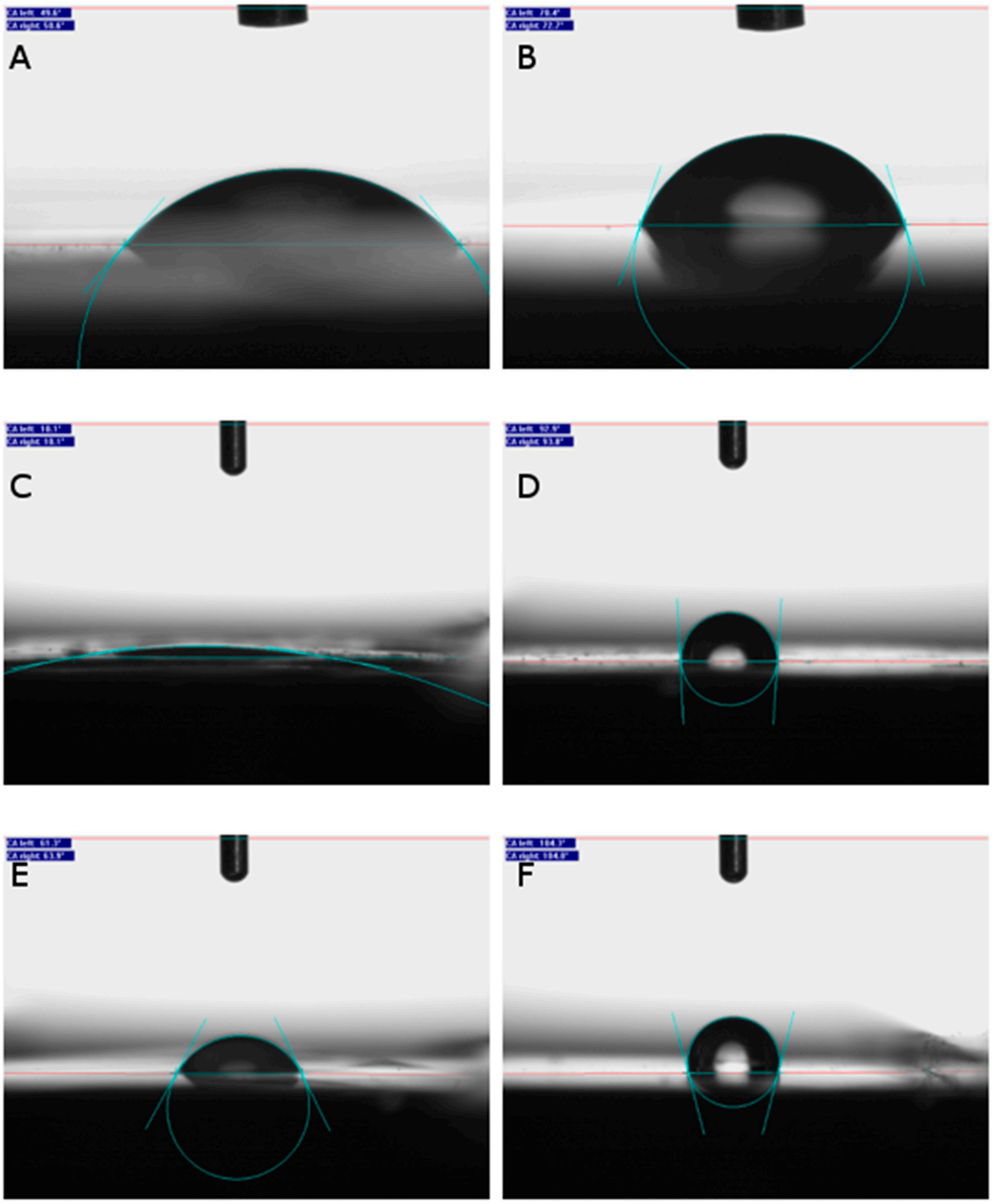

Figure S2: Exemplary photographs of contact angle measurements on track-etched membranes. A) Base membrane, 0.2 µm pore size, B) base membrane, 1 µm pore size C) PAGA-functionalised membrane 0.2 µm pore size D) PAGA-functionalised membrane 1 µm pore size, D) PNADha-functionalised membrane 0.2 µm pore size D) PNADha-functionalised membrane 1 µm pore size.

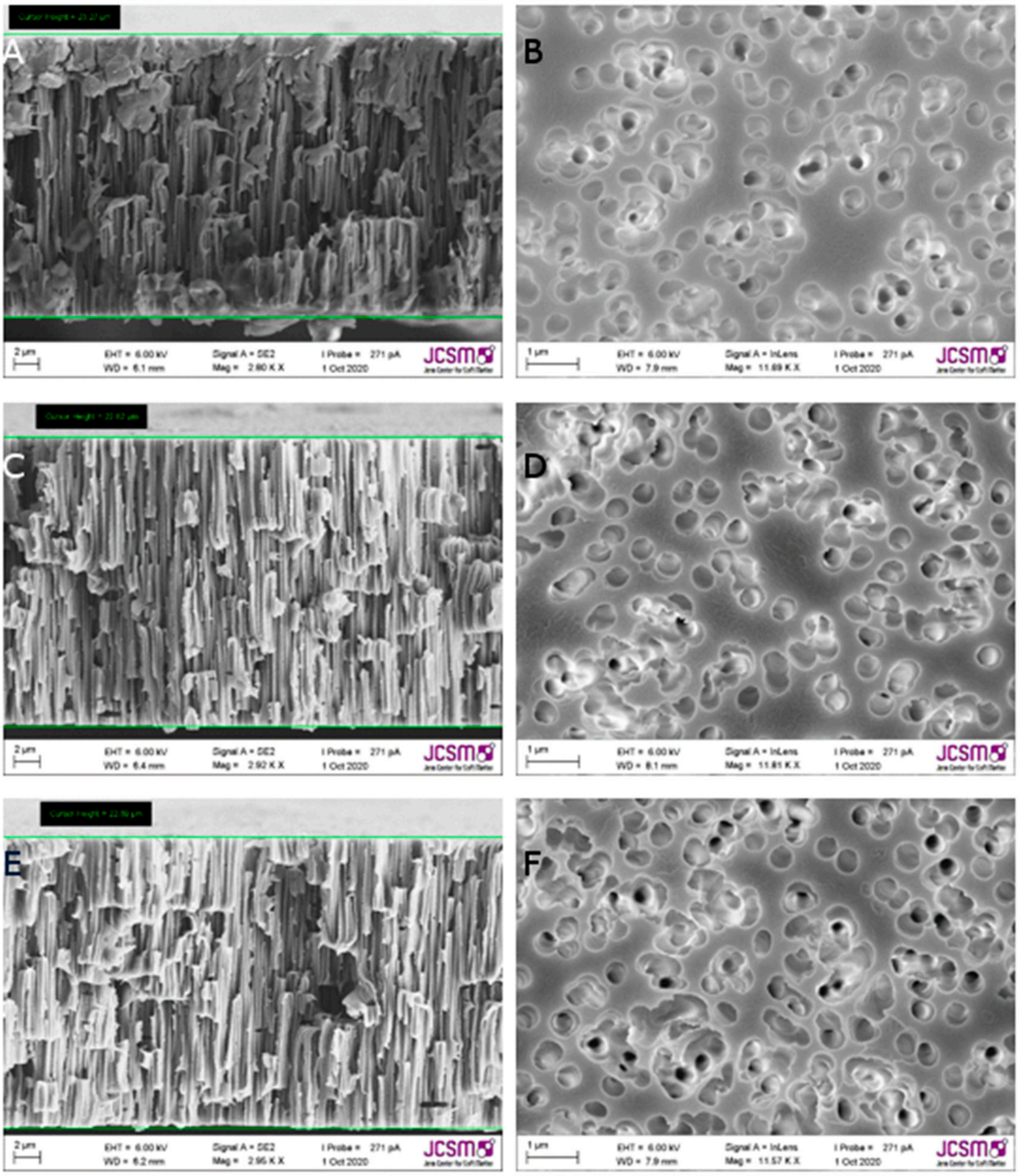

Figure S3: SEM micrographs of membranes with a nominal pore size of 0.2 µm. A) Base membrane: cross section, B) Base membrane: top view, C) PAGA-functionalised membrane: cross section, D) PAGA-functionalised membrane: top view, E) PNADha-functionalised membrane: cross section, F) PNADha-functionalised membrane : top view.

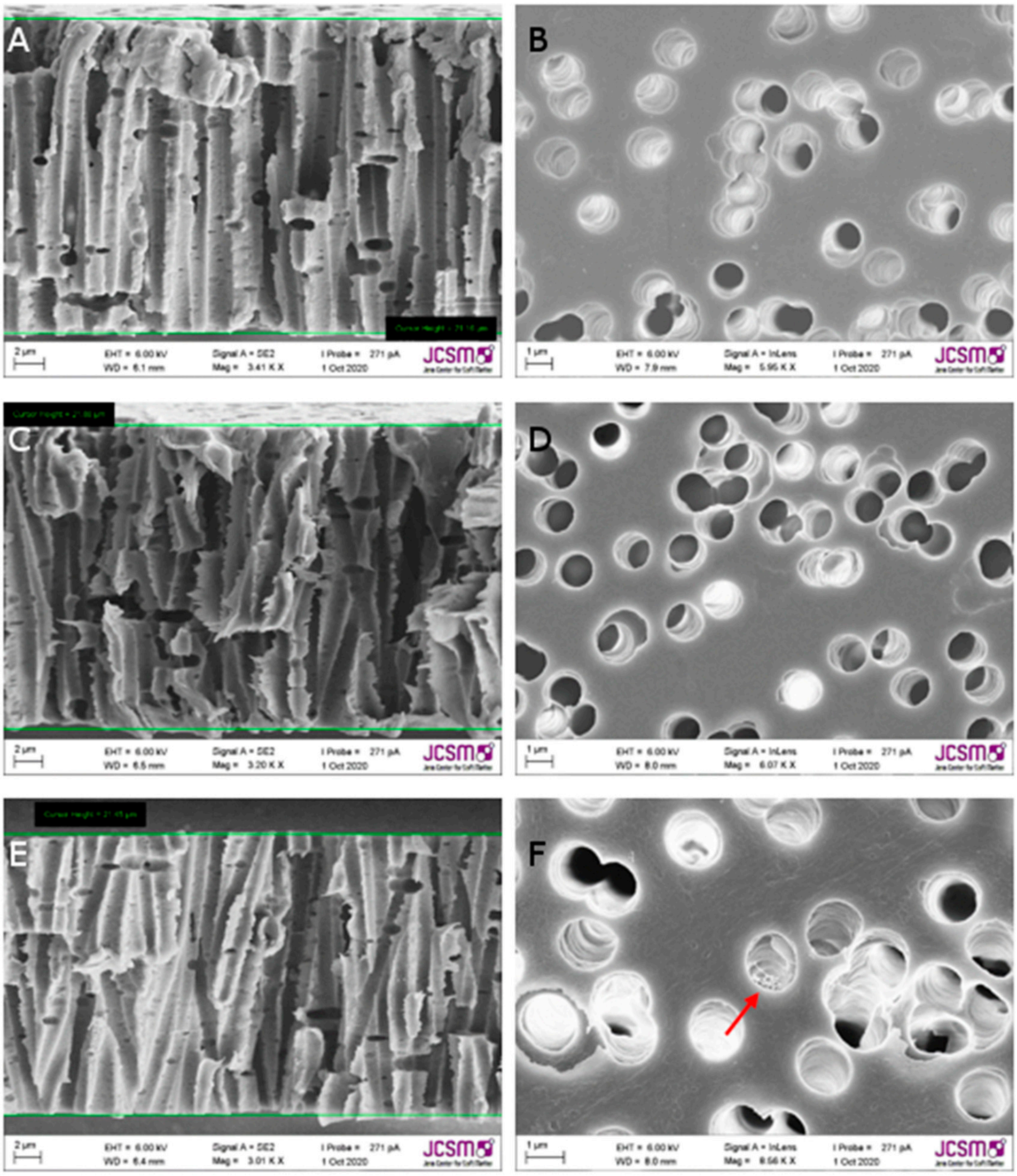

Figure S4: SEM micrographs of membranes with a nominal pore size of 1 µm. A) Base membrane: cross section, B) Base membrane: top view, C) PAGA-functionalised membrane : cross section, D) PAGA-functionalised membrane: top view, E) PNADha-functionalised membrane: cross section, F) PNADha-functionalised membrane: top view.

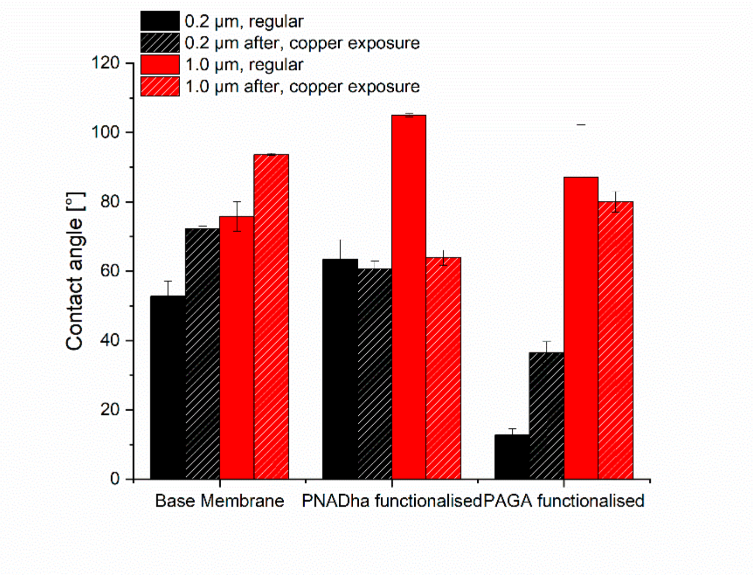

Figure S5: Changes in the contact angle of the membranes with water upon exposure to an aqueous solution of Cu(NO3)2 (0.4 M).

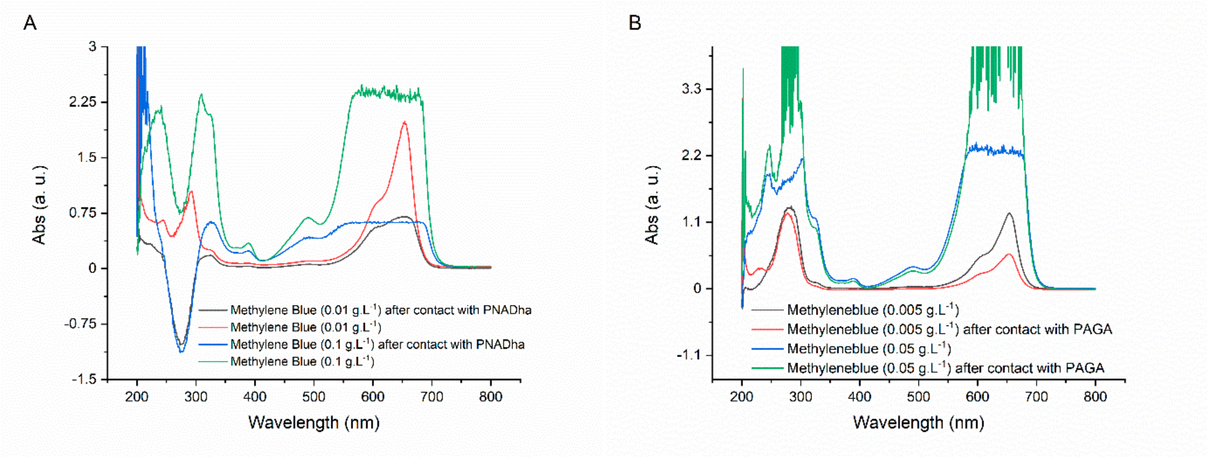

Figure S6: UV/Vis absorption spectra for solutions methylene blue in ethanol before and after the contact with A) PNADha and B) PAGA.
